# Normocaloric Low Cholesterol Diet Modulates Th17/Treg Balance in Patients with Chronic Hepatitis C Virus Infection

**DOI:** 10.1371/journal.pone.0112346

**Published:** 2014-12-22

**Authors:** Roberta Maggio, Carmela Viscomi, Paola Andreozzi, Gabriella D'Ettorre, Giovanni Viscogliosi, Barbara Barbaro, Manuele Gori, Vincenzo Vullo, Clara Balsano

**Affiliations:** 1 Laboratory of Molecular Virology and Oncology, Francesco Balsano Foundation, Rome, Italy; 2 Predictive Medicine Unit, Sapienza University, Rome, Italy; 3 Department of Public Health and Infectious Diseases, Sapienza University, Rome, Italy; 4 Institute of Molecular Biology and Pathology, National Research Council, Rome, Italy; Università degli Studi di Palermo, Italy

## Abstract

Hepatitis C virus (HCV) infection is associated with hepatic and extrahepatic manifestations, including immunological disorders. Chronic Hepatitis C (CHC) is often characterized by cholesterol and lipid metabolism alterations, leading to hepatic steatosis. Cholesterol metabolism, in fact, is crucial for the viral life cycle. Recent works described that a higher dietary cholesterol intake is associated with the progression of HCV-related liver disease. CHC patients have increased levels of T helper 17 (Th17)-cells, a lymphocytic population involved in the pathogenesis of liver inflammation and autoimmune hepatitis. The balance between Th17 and regulatory T (Treg) cells is crucial for chronic inflammation and autoimmunity. Th17-cell differentiation is deeply influenced by the activation LXRs, nuclear receptors modulating cholesterol homeostasis. Moreover, HCV may affect these nuclear receptors, and cholesterol metabolism, through both direct and indirect mechanisms. On these bases, we hypothesized that modulation of cholesterol levels through Normocaloric Low Cholesterol Diet (NLCD) may represent an innovative strategy to reduce the progression of HCV infection, through the modulation of peripheral Th17/Treg balance. To this end, we performed a pilot study to investigate whether a Normocaloric Low Cholesterol Diet may be able to modulate Th17/Treg balance in patients affected by chronic HCV infection. After 30 days of NLCD CHC patients showed a significant reduction in Th17 cells frequency, which correlated with strong reduction of IL-17 and IL-22 serum levels. At the same time, we appreciated an increase in the percentage of Treg cells, thus improving Treg/Th17balance. Moreover, we observed an increased expression of LXRs and their target genes: SREBP-1c and ABCA-1. In conclusion, NLCD finely regulates Th17/Treg balance, improving immune system response in CHC patients. This study could pave the way for new treatments of CHC patients, suggesting that change in lifestyle could support the management of these patients, promoting well-being and possibly hindering disease progression.

**Trial Registration:**

ClinicalTrials.gov NCT02038387

## Introduction

Hepatitis C virus (HCV) infection affects about 170 million people worldwide; the acute phase of infection is rarely cleared and most of patients become chronically infected. Chronic infection by HCV is often characterized by lipid metabolism disorders that lead to hepatic steatosis [1]. In addition, lipid metabolism and cholesterol have a key role in viral life cycle [2]. In particular, plasma membrane cholesterol is required for HCV entry [3]; moreover, HCV replication takes place in cholesterol rich domains within the viral replication complex [4]. Recently, higher dietary cholesterol intake was associated with the progression of HCV related liver disease progression [5]. Therefore, dietary modulation of cholesterol intake may represent an innovative strategy to reduce the progression of HCV infection.

HCV usually induces robust immune responses, however it frequently escapes the immune defense to establish persistent infection [6]. Although the underlying mechanisms for HCV persistence and disease pathogenesis are not fully understood, a role for interleukin (IL)-17-producing CD4^+^ T cells, also named T-helper 17 cells (Th17), has been proposed [7], [8]. Th17 cells produce IL-17A together with other cytokines, such as IL-17F, IL-21, and IL-22, and express CD161, that gives to these cells a specific liver homing phenotype [9]–[11]. In particular, Th17 cells have been described as the principal mediators not only in autoimmune diseases but especially in chronic inflammatory disorders[12]. Several authors described an increased amount of circulating and intrahepatic Th17 cells in Chronic Hepatitis C (CHC) patients which correlates with the severity of liver inflammation [13]–[15]. Notably, the development of Th17 cells is reciprocally interconnected with that of regulatory T cells (Treg) [16]. Th17 cells represent a pro-inflammatory subset, which when in excess contributes to autoimmunity and tissue damage, whereas Treg cells have an antagonistic effect, which when in failure also contributes to the same diseases. Hence, Treg and Th17 cells arise in a mutually exclusive fashion, and a balance between the two T-cell subsets is crucial for the immune homeostasis [17]–[19].

Furthermore, recent studies highlight that Th17 cell-differentiation can be regulated by nuclear receptor LXRs (Liver X Receptors), known as LXRα and LXRβ [20], [21]. These nuclear receptors act as important modulators of lipid metabolism and cholesterol homeostasis by regulating genes, such as SREBP-1c (sterol regulatory element-binding protein 1c), and ABCA-1 (ATP-binding cassette transporter-1) [22]. Therefore, considering the importance of cholesterol for the life cycle of the HCV [2], and the involvement of LXRs in the modulation of the immune response, we thought that cholesterol [23], [24], via LXRs-mediated signaling [23], [24], could represent a key element in regulating the differentiation of T lymphocytes in Th17 cells. In addition, CHC patients have high serum levels of oxysterols, endogenous ligands of LXRs and products of cholesterol oxidation [25]. Furthermore, it has been reported a direct interaction between HCV-core protein and Retinoid X Receptor (RXR) alpha [26], [27], a well-known heterodimeric partner of LXRs. So the RXR/HCV-core complex might deregulate the LXRs activity during HCV infection, supporting the influence of HCV on LXRs and in turn on the frequency of Th17 cells, thus potentially affecting the host immune system.

On these bases, we performed a pilot study to investigate if a Normocaloric Low Cholesterol Diet (NLCD) may be able to modulate Th17/Treg balance in patients affected by chronic HCV infection. With this experimental setting we directly compared the effect of NLCD in CHC patients compared to nonalcoholic fatty liver disease (NAFLD) and non-alcoholic steatohepatitis (NASH) patients, since the last two categories of patients are characterized by both hepatic steatosis and lipid disorders [28], similar to the ones observed in CHC patients, but in absence of the viral involvement.

## Patients and Methods

### Ethics Statement

The protocol for this trial and supporting checklist are available as supporting information; see S1 CONSORT Checklist and S1 Protocol. The study was approved by the local Ethics Committee of the Sapienza University, Rome, Italy, (approval number: 2534; on July 26,2012) and is conducted according to the principles expressed in the Declaration of Helsinki. The trial is registered on the ClinicalTrials.gov (registration number: NCT02038387). This study was recorded after enrolment of participants started, because it is a purely observational study which does not include drug administration; thus, in the first instance, we did not consider required to record it at ClinicalTrials.gov. Moreover, we have adhered exactly to the procedures approved by the Institutional Ethics Committee, on July 26, 2012, which has been obtained before the beginning of the trial. The first recruitment of patient was performed in 2013, and recruitment of new patients is still ongoing. The authors confirm that all ongoing and related trials for this intervention are registered. For each patient the follow-up duration is 30 days. All participants gave written informed consent.

### Patients

**Figure 1:**
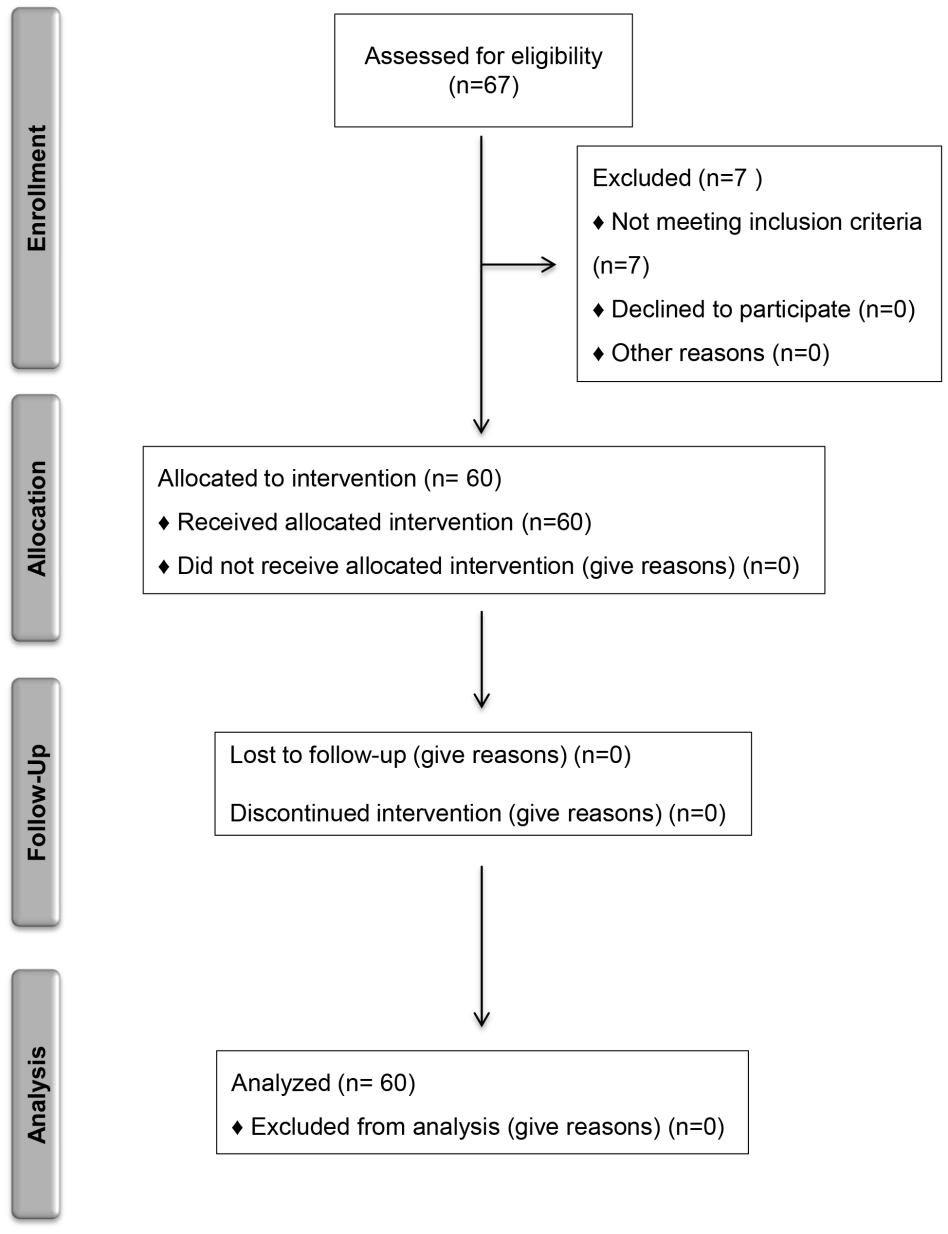
CONSORT Flow Diagram.

Thirty CHC patients and 30 NAFLD/NASH patients, diagnosed by ultrasonography [29] [7% of them had NASH proven by biopsy], underwent the dietary regimen (Fig. 1). No patients received any pharmacological treatment at least 6 months before entering the study, in particular drugs potentially affecting cholesterol metabolism (e.g. statins, phytosterols, etc.). We excluded cirrhotic patients. Others exclusion criteria were as follows: co-infection by hepatitis B virus, or human immunodeficiency virus infections, autoimmune diseases, and other relevant associated-diseases such as decompensated diabetes, kidney diseases, pulmonary diseases, tumors.

The patients were instructed to follow a Normocaloric Low Cholesterol Diet (1400 Kcal for females and 1800 Kcal for males) for a period of 30 days. Dietary compounds were distributed as follow: lipids 23% (cholesterol 185 mg/day), carbohydrates 50%, and proteins 27%. The salt intake was maintained to what that patients had previously, to do not to influence the frequency of Th17 cells based on the content of salt [30].

Dietary habits, before the study, were recorded for all enrolled patients, finding no considerable differences in nutrient intake between the CHC patients and the control group (NAFLD/NASH subjects).

The patients were instructed to follow the same food recipe for meal preparation. The diet was provided by an expert nutritionist. Adherence to the diet was monitored by phone interview with a weekly frequency. Subjects were advised to maintain their normal living activity and sleeping patterns during the intervention period. Subjects were instructed to follow the diet on the day after baseline measurements (day 1). At the beginning (day 0, namely T0) and end (day 30, namely T30) of diet, blood samples were analyzed for general parameters including biochemical parameters, hematology, and coagulation values (Table 1). NLCD is not provided to reduce the patient weight, but rather to influence the virus activity, known to be dependent by cholesterol.

**Table 1:** Clinical and biochemical characteristics of studied patients before and after NLCD.

	CHC pts (n = 30)			NAFLD/NASH pts (n = 30)		
Variable	T0	T30	*p-value*	T0	T30	*p-value*
Sex (F/M)	13/17			12/18		
Age (years)	57.7±12.3			57.5±12.1		
Body weight (kg)	73.6±10.1	71.2±9.7	NS	82.2±21.5	80.8±22.8	NS
BMI (kg/m^2^) (≤25)	26.3±4.1	25.8±3.7	NS	29.7±6.1	28.8±6.5	NS
Systolic blood pressure (<130 mmHg)	137.1±17.3	129.5±12.6	<0.05	136.7±6.4	129.8±12.4	<0.05
Diastolic blood pressure (<80 mmHg)	83.3±11.3	76.5±8.9	<0.05	80.3±11.0	75.9±8.9	<0.05
HOMA-IR (<2)	3.0±2.0	2.8±1.2	NS	3.1±1.9	3.0±1.9	NS
Total cholesterol (81.1–235.5 mg/dL)	156.3±26.0	141.0±21.0	NS	178.2±45.5	174.0±41.0	NS
HDL cholesterol (34.7–56 mg/dL)	54.1±15.2	54.1±13.1	NS	52.5±14.5	52.9±21.9	NS
LDL cholesterol (54.8–120 mg/dL)	91.5±32.1	80.1±45.4	<0.001	109.4±40.2	94.5±41.5	<0.01
Triglycerides (45.1–235 mg/dL)	112.6±22.1	107.1±52.8	NS	116.4±44.3	115.2±48.2	NS
ALT (10–45 UI/mL)	60.2±37.3	35.5±13.7	<0.05	53.9±42.9	53.5±43.3	NS
AST (9–40 UI/mL)	75.1±59.4	66.8±49.4	<0.05	41.5±32.5	37.7±31.2	NS
GGT (8–61 UI/mL)	44.6±33.8	42.1±29.4	<0.05	50.1±49.8	36.0±22.9	<0.05
AP (40–129 UI/mL)	92.1±20.2	65.3±13.6	<0.05	76.5±36.5	69.2±31.1	NS
hsCRP (0.1–0.6 g/L)	0.9±0.1	0.4±0.1	<0.05	0.6±0.1	0.4±0.1	NS
Bilirubin (<1.20 mg/dL)	0.53±0.1	0.49±0.1	NS	0.62±0.3	0.60±0.3	NS
Uric acid (<7.2 mg/dL)	5.1±1.3	4.5±1.2	<0.05	5.0±1.3	4.6±1.3	<0.05
HCV RNA (log IU/mL)	1.9± 0.2	1.7± 0.9	NS			
Fibroscan (0–36 kPa)	9.1±0.5	6.9±0.8	NS	10.1±0.5	7.0±0.9	NS
F0 (0 kPa)	49.3%			F0: 57.3%		
F1 (0–8.5 kPa)	35.1%			F1: 38.6%		
F2–3 (8.6–13 kPa)	15.6%			F2: 4.1%		
HCV genotype	1a 37%, 1b 24%, 2a/2c 39%					

T0 =  Before NLCD; T30 =  After NLCD; Pts =  Patients; F = Females; M = Males; BMI =  Body max index; HOMA-IR = Homeostatic model assessment-insulin resistance; HDL =  High-density lipoprotein; LDL = Low density lipoprotein; ALT = Alanine transaminase; AST = Aspartate transaminase; GGT = Gamma-glutamyl transferase; AP = Alkaline phosphatase; hsCRP = high-sensitivity C-reactive Protein. Statistical significance was accepted at *p*<0.05; NS = Not significant. Values are presented as mean± SD.

### Cell isolation and stimulation

On day 0 and day 30, peripheral blood mononuclear cells (PBMCs) were isolated from heparinized blood samples by Ficoll-Hypaque (Eurobio, Les UlisCedex, France) density-gradient centrifugation. Mononuclear cells were resuspended in RPMI (Cambrex BioScience, Verviers, Belgium) completed with 10%heat-inactivated fetal calf serum (FCS, HyClone, South Logan, UT) and 1%glutamine (all from Euroclone, Milan, Italy), and left stimulated with 50 ng/ml Phorbol Myristate Acetate (PMA, Sigma-Aldrich, St Louis, MO), 1 µg/ml Ionomycin (Iono, Sigma-Aldrich). The stimulation was carried out at 37 °C for 5 h, in the presence of the intracellular trafficking inhibitor Brefeldin A (10 µg/ml BFA; Sigma-Aldrich), before intracellular staining and cytofluorimetric analysis.

### Intracellular staining and multiparameter cytofluorimetric analysis

After stimulation, cells were washed twice in Ca^++^/Mg^++^-free phosphate-buffered saline (PBS, Cambrex BioScience) and subsequently treated using the Fix and Perm cell permeabilization kit (Caltag Laboratories, Burlingame, CA), according to the manufacturer's instructions. For Th17-cell examination, PBMCs were first surface-stained with fluorochrome-conjugated anti-CD161, CD3, CD4, and isotype-matched control mAb were purchased from BD Biosciences (San Jose, CA, USA), for 20 min at 4°C. Then the cells were fixed and permeabilized, and stained intracellularly with phycoerythrin (PE)-conjugated anti- IL-17Aand anti-IL-22 PE (all eBioscience, San Diego, CA, USA).

For Treg-cell examination, PBMCs were firstly surface-stained with fluorescein isothiocyanate(FITC)-conjugated anti-human CD4 antibodies, Alex Fluor 647-conjugated anti-human CD127 antibodies, peridinin chlorophyll protein (PerCP)-conjugated anti-human CD3 antibodies and allophycocyanin (APC)-conjugated anti-human CD25 antibodies for 30 min, then lysed with FACSTM lysing solution (BD PharMingen, San Jose, CA), and treated with fix/perm mixture, according to the manufacturer's instructions. Finally, cells were incubated with PE-conjugated anti-human Foxp3 antibodies overnight. Isotope controls were used to ensure antibody specificity.

Stained cells were acquired with an LSR Fortessa flow cytometer (Becton Dickinson) and analyzed with FACSDiva software (BD Biosciences) or FlowJo software (Treestar, Ashland, OR). Lymphocyte region was defined on the basis of forward and side scatter physical parameters; T helper and Treg cell subsets were identified by anti-CD3 and anti-CD4 staining. Samples were gated on live, single CD3^+^T lymphocytes.

### Real Time qPCR

Total RNA was isolated from patients' PBMCs using the Trizol reagent (Invitrogen, by Life Technologies), and 2 µg of complementary DNA was prepared using Random Primers, and AMV Reverse Transcriptase of the Reverse Transcription System (A3500, Promega) according to the manufacturer's protocol. PCR primers were designed using AmplifX 1.5.4 software, and NCBI Primer-BLAST and were as follows: human LXRα, *forward* 5′-TGCCGAGTTTGCCTTGCTCATT-3′ and *reverse* 5′-GGAGGCTCACCAGTTTCATTAGCA-3′; human LXRβ *forward* 5′-AAGAAGGGCCCAGCCCCGAA-3′ and *reverse* 5′-ACACTGCGCCGGAAGAAGCC-3′; human SREBP-1c, *forward* 5′-TTCCGCCCTTGAGCTGCGTG-3′ and *reverse* 5′-CCGGAAGCTCTGTGCCAGCC-3′; human ABCA-1, *forward* 5′-CTGGGCCACAATGGAGCG-3′ and *reverse* 5′-ATGTAGGCGGTGCCCGAGGT-3′. Primers for β-actin were used as housekeeping controls: human β-actin, *forward* 5′-GCACTCTTCCAGCCTTCC-3′ and *reverse* 5′-AGGTCTTTGCGGATGTCCAC-3′. QRT-PCR was performed with 7500 Fast Real-Time PCR System (Applied Biosystems, CA, USA) using Power SYBR Green PCR Master Mix as the fluorophore, including ROX as passive reference (Applied Biosystems). All PCR conditions and primers were optimized to produce a single product of the expected base pair size. All the experiments were performed in triplicate.

### Enzyme-Linked Immunosorbent Assay (ELISA)

Serum concentration of IL-17, IL-22, IL-23 (R&D Systems, Minneapolis, MN, USA), and TGF-β (eBioscience) were measured by commercially available ELISA kits, according to the protocols provided by the manufacturer. To determine the serum level of hyaluronic acid (HA) we used a quantitative test kit (Corgenix, Denver, CO). All the samples were assessed in triplicate.

### Statistical analysis

Results are expressed as means ± standard deviation (SD) for raw data and as percentages ± SD. We performed a paired Student *t*-test comparing T30 vs T0 time point within the same group of patients analyzed. Correlation analysis was performed using Pearson correlation test. Sample size was determined on the basis of recent studies [7], [14], [15] supporting the hypothesis that the Th17 cell frequency, the variable in our cohort of patients, has likely a mean of 3.2%±2.0. We hypothesized that diet can decrease the Th17 cell frequency to 2.0%±0.15, power = 90%, significance level  = 0.05; for this reason to perform the study, at least 24 patients per group are required. Thus, we enrolled 30 patients per group considering the possible dropout of about the 20% of them. All statistical analyses were performed using PRISM v.5 software (GraphPad Software, San Diego, CA); *p*-value <0.05 was considered statistically significant.

## Results

### Clinical parameters of CHC and NAFLD/NASH patients after NLCD

Thirty CHC and 30 NAFLD/NASH patients were instructed to follow a NLCD for 30 days. We didn't observe any significant difference of the actual adherence to the diet among the two groups of patients studied. Before and after the dietetic treatment, the anthropometric characteristics and biochemical parameters were assessed (Table 1 and Fig. 1).

In CHC patients liver fibrosis, evaluated by biopsies and Fibroscan, showed the following distribution: 49.3% F0, 35.1% F1, and 15.6% F2–3. HCV genotypes were distributed as follow: 37% 1a, 24% 1b, and 39% 2a/2 c. Liver fibrosis in NAFLD/NASH patients showed the following distribution: 57.3% F0, 38.6% F1, and 4.1% F2. Liver biopsies of CHC and NASH patients showed evidence of an average of 20–30% and 40–50% of macrovacuolar steatosis of the hepatocytes, respectively.

In our cohorts of patients, we found, as expected, a statistically significant reduction of low-density lipoprotein (LDL) cholesterol (*p*<0.001 and *p*<0.05, respectively), after NLCD. Only CHC displayed a reduction tendency for total cholesterol and triglycerides, without reaching statistical significance. Liver damage, after NLCD, resulted significantly improved in CHC patients. In fact, aspartate aminotransferase (AST) and alanine aminotransferase (ALT), gamma-glutamyltransferase (GGT) and alkaline phosphatase concentrations were significantly lower in treated patients compared to the basal conditions (*p*<0.05); whereas, after diet, NAFLD/NASH patients showed a significant statistical reduction only for GGT serum levels (*p*<0.05).

### Impact of NLCD on peripheral IL-17- and IL-22-producing CD161^+^CD4^+^T cells, defined as Th17 cells

Firstly, we investigated the percentage of Th17 cells in the peripheral blood of the CHC and NAFLD/NASH patients. Our experiments demonstrated and confirmed a significant increase in frequency of IL-17- and IL-22-secreting Th17 cells in CHC and NAFLD/NASH patients compared to healthy donor [13]–[15] (data not shown).

**Figure 2:**
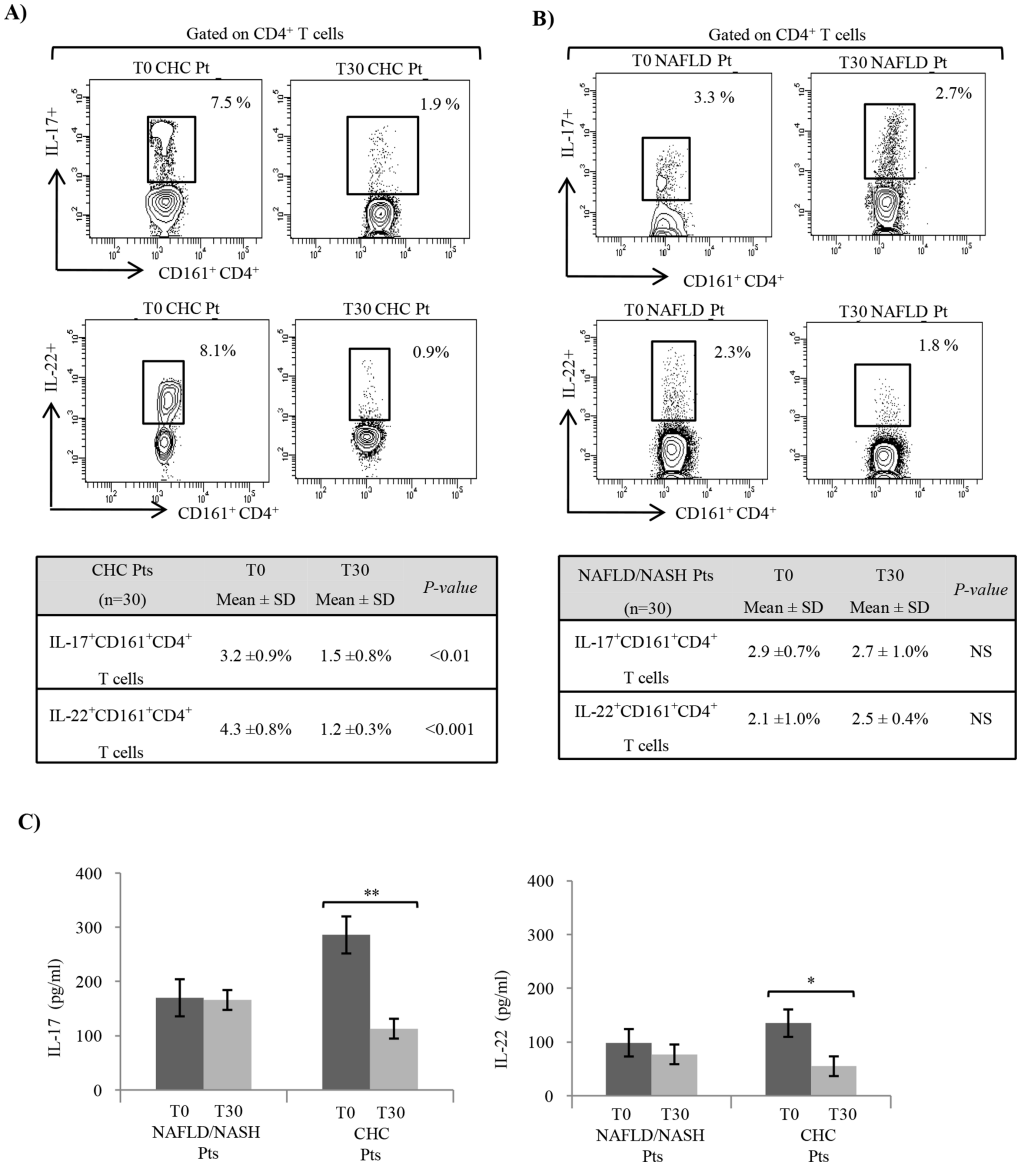
IL-17^+^and IL-22^+^CD161^+^CD4^+^T-cellphenotype in CHC and NAFLD/NASH patients before and after NLCD. A) Before (T0) and after (T30) NLCD, in CHC patients, we observed a statistically significant decrease of IL-17- and IL-22-producing cells among the indicated CD161^+^CD4^+^T fractions and gated live CD4^+^CD3^+^ lymphocytes. B) These changes in frequency of IL-17^+^ and IL-22^+^Th17 cells did not reach statistical significance before (T0) and after (T30) NLCD in NAFLD/NASH patients. C) After diet in CHC and NAFLD/NASH patients, we observed the same trend of serum levels of IL-17 and IL-22, even if no statistical significance was reached in NAFLD/NASH patients. One representative plot of all independent experiments is shown. *P<0.05; **P<0.01; ***P<0.001.

After 30 days of NLCD, in CHC patients we detected a statistically significant reduction in the frequency of IL-17- and IL-22-producing Th17 cells (Fig. 2A). Otherwise, in NAFLD/NASH patients we did not found any significant difference in Th17-cell frequency before and after diet (Fig. 2B). Th17 related cytokine were also assayed before and after diet in serum of both groups of patients. Serum levels of IL-17 and IL-22 were significant decreased only in CHC patients (Fig. 2C). The same trend after diet we observed for the serum levels of Th17-related cytokines, even if no statistical significance was reached (Fig. 2C).

Moreover, in CHC but not in NAFLD/NASH patients, both before and after NLCD, the Th17-cell frequency positively correlated with ALT levels (r = 0.445, *p*<0.001, vs r = 0.305, *p*<0.001). No relationship was highlighted between theTh17-cell modulation by NLCD and the HCV-RNA levels or the different HCV genotypes (data not shown).

### Frequency of peripheral Treg cells and Treg/Th17 ratio after NLCD

**Figure 3:**
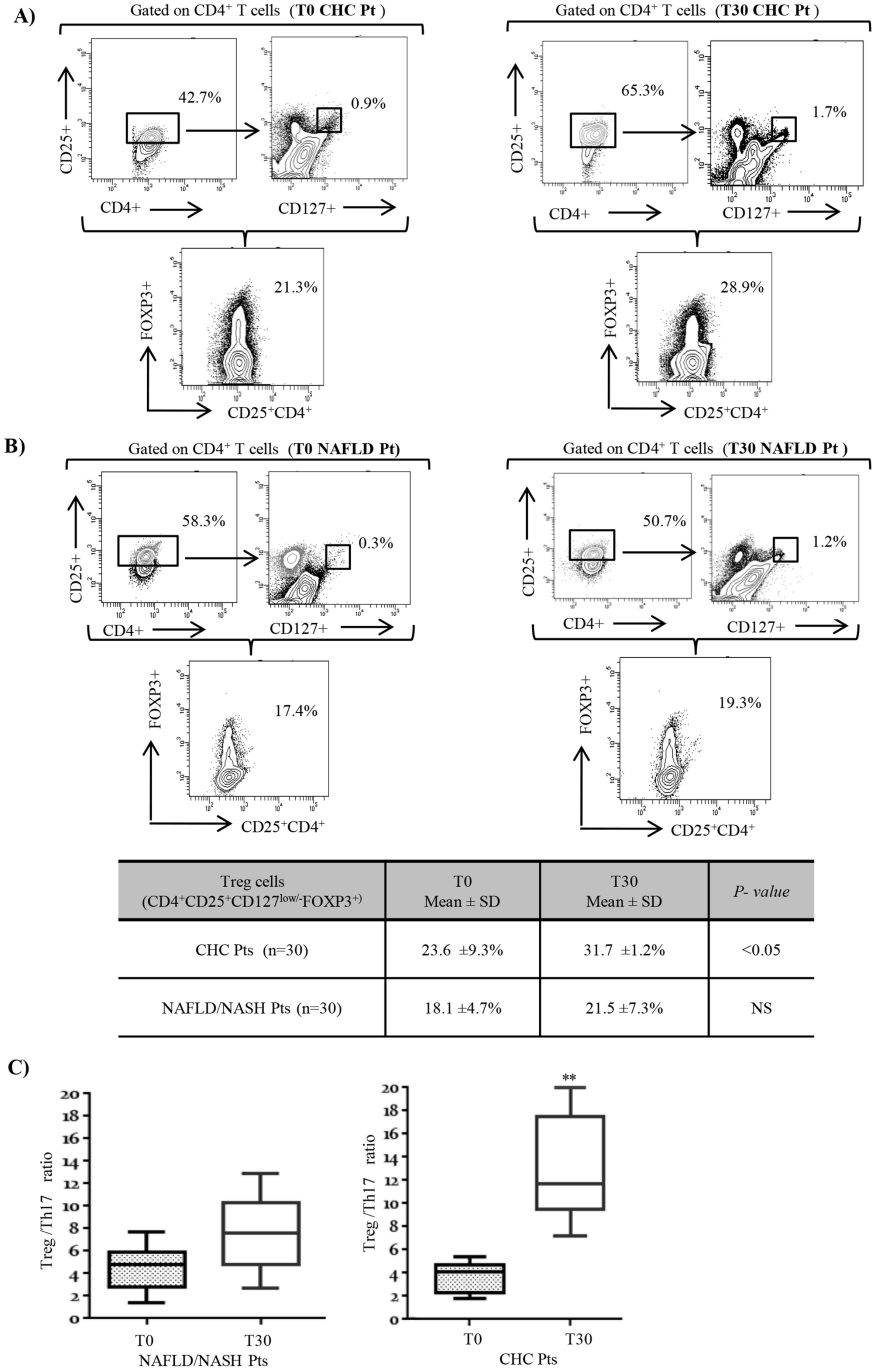
Change of the Treg-cell frequency and Treg/Th17 ratio in NAFLD/NASH and CHC patients, before and after NLCD. Evaluation of Treg cells by flow cytometry was determined as percentage of CD127low/-CD25+FoxP3+ cells within the CD3+CD4+compartment. We reported the dot plots of representative experiments, with the percentage of CD25+CD4+ Treg cells, which CD25+ are CD127 low or negative, and the percentage of CD4+CD25+CD127low/-FOXP3+ Treg cells before and after NLCD in peripheral blood of CHC (A) and NAFLD/NASH patients (B). In table, we reported a statistically significant difference between T0 and T30 in Treg-cell frequency in CHC, but not in NAFLD/NASH patients. In C) We represents the changes in ratio between CD4+CD25+CD127low/-FOXP3+ Treg and Th17 (IL-17^+^CD161^+^CD4^+^ T) cells frequencies obtained from both groups studied before and after NLCD. Horizontal bars represent the median values of indicated index. *P<0.05; **P<0.01.

We analyzed the basal Treg-cell frequency in peripheral blood of CHC and NAFLD/NASH patients. Treg cells are a CD4^+^CD3^+^ T lymphocyte population expressing, low levels of CD127 and high levels of CD25 together with the forkhead box/winged helix transcription factor P3 (FoxP3). Thus, in the lymphogate CD3^+^CD4^+^ cells were detected the Treg cells by combination of CD25+CD127low/- and FOXP3+ [31]. The percentage of this circulating cellular population resulted more elevated in CHC patients after NLCD (Fig. 3A), resulting in a considerable improvement in the Treg/Th17 ratio (*p*<0.01, Fig. 3C). No changes were observed in NAFLD/NASH patients (Fig. 3B,C), supporting the hypothesis that these findings were specific for HCV infection. No clear correlation between Treg/Th17 ratio, viral load and different HCV genotypes was highlighted (data not shown).

### LXRs and related genes in CHC and NAFLD/NASH patients after NLCD

In order to understand whether NLCD is able to modulate the LXRs activity, we assayed the gene expression profiling of the LXRs and their target genes, in PBMCs of both CHC and NAFLD/NASH patients.

**Figure 4:**
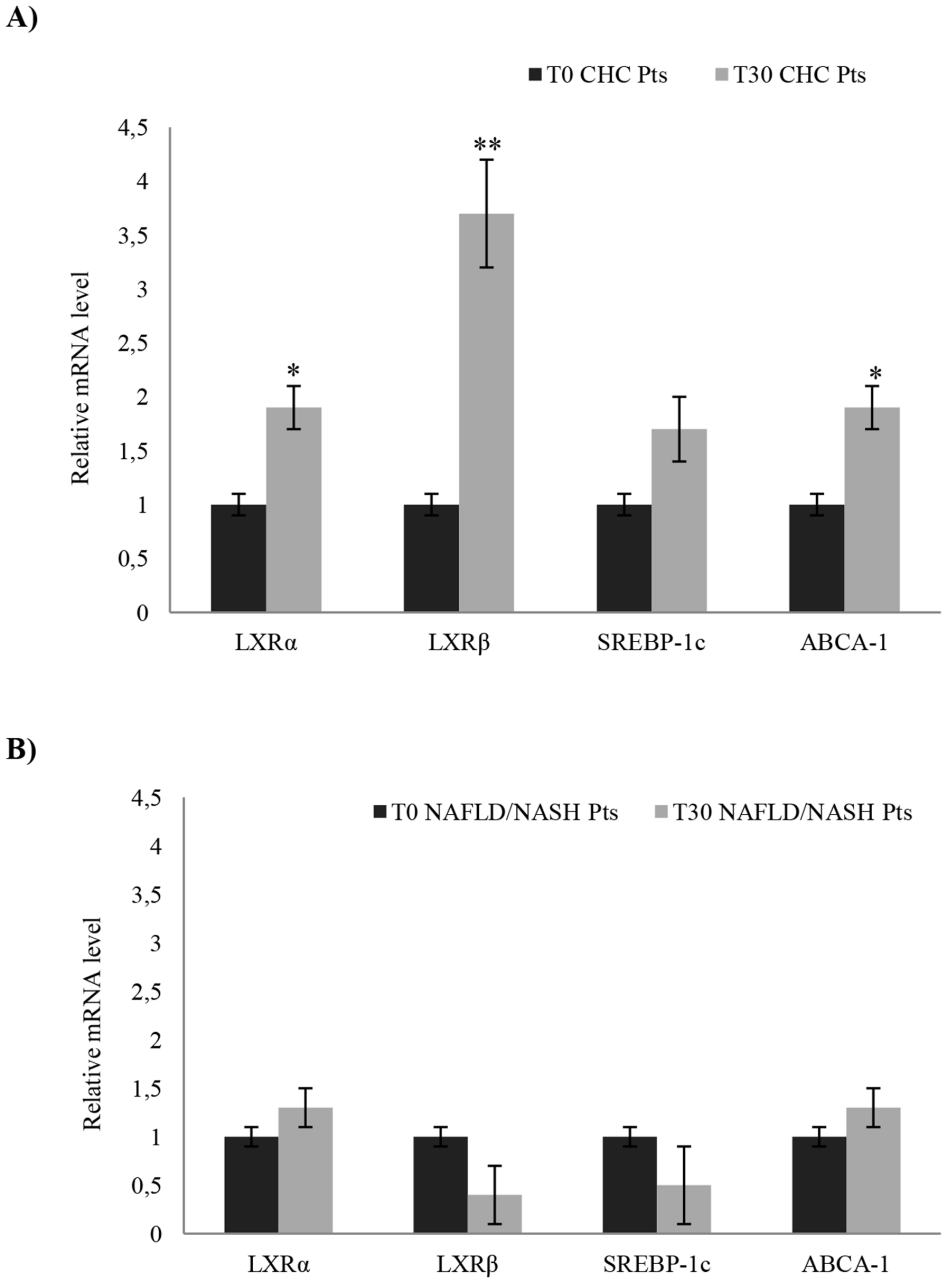
Relative mRNA expression of LXRs and related-gene obtained from PBMCs of NAFLD/NASH and CHC patients, after NLCD. In CHC patients, after NLCD, we observed highly significant changes in LXRα, LXRβ and ABCA-1 mRNA levels, respect to before NLCD (A). No significant changes were observed in NAFLD/NASH patients after NLCD (B). Data are expressed as mean ±SD; all samples were assessed in triplicate. Error bars illustrate SD. *P<0.05; **P<0.01.

The LXRs mRNA expression levels, in particular of the LXR β, were significantly increased in CHC patients after NLCD (Fig. 4 A). Then, we analyzed two LXR-target genes, ABCA-1 and SREBP-1c, involved in the regulation of cellular cholesterol efflux and phospholipids homeostasis. We observed increased levels of ABCA-1 and SREBP-1c mRNA after NLCD (Fig. 4 A), suggesting a functional activation of LXRs in CHC patients. Conversely, in NAFLD/NASH patients, we observed an opposite trend in the modulation of LXRs and related genes by diet, with respect to CHC patients (Fig. 4 B).

### Effect of NLCD on pro-inflammatory cytokines in CHC and NAFLD/NASH patients

**Figure 5:**
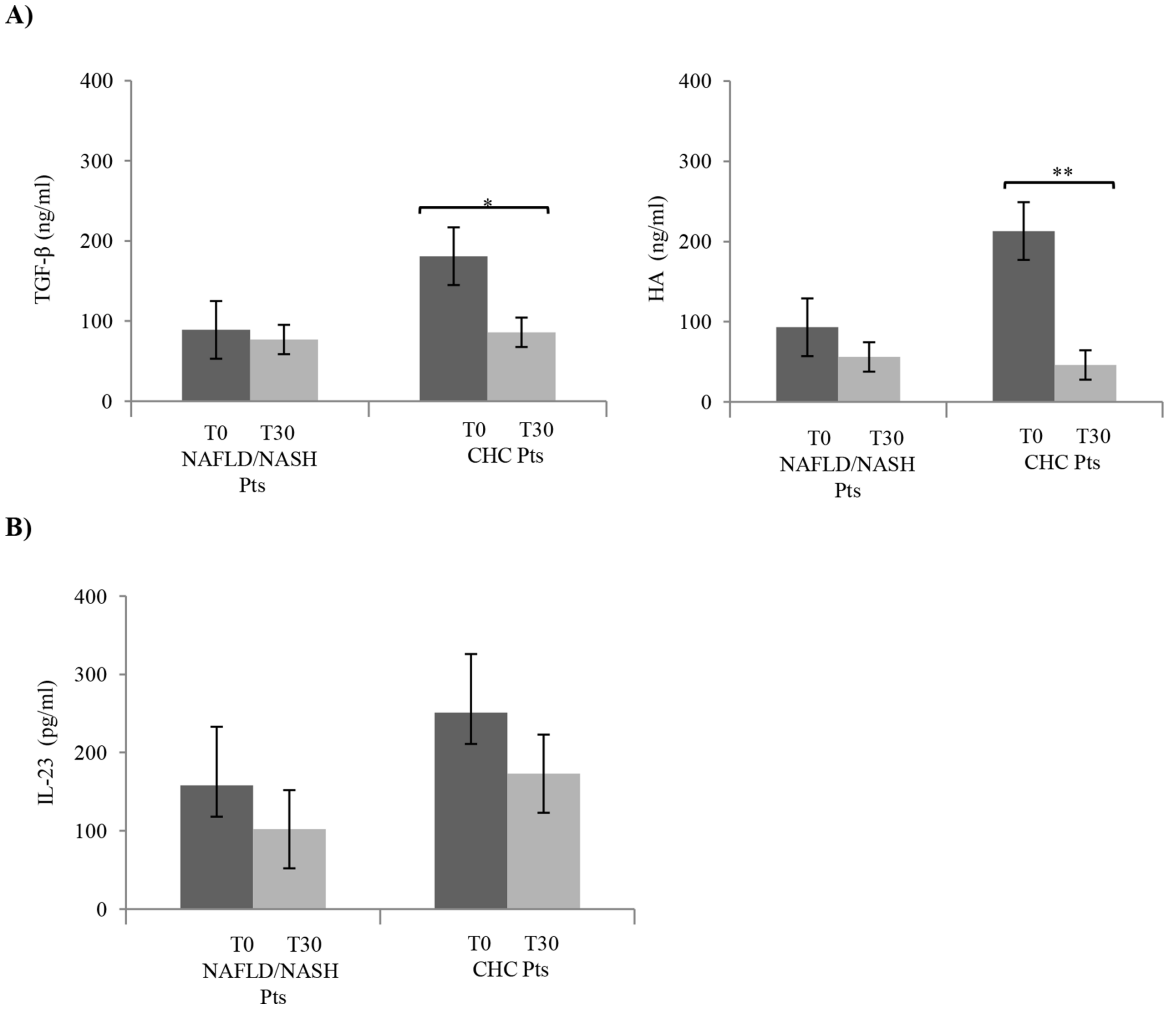
Serum levels of (A) TGF-β, HA and (B) IL-23 obtained from NAFLD/NASH and CHC patients before and after NLCD. Serum levels of pro-inflammatory cytokines were lowered by NLCD in CHC and NAFLD/NASH patients. Data are expressed as mean ±SD; all samples were assessed in triplicate. Error bars illustrate SD. *P<0.05; **P<0.01.

We determined the effect of the low cholesterol diet on the transforming growth factor-beta (TGF-β) serum levels; this cytokine was analyzed because it is implicated in Th17-cell differentiation and also because it is considered a biomarker of chronic inflammatory processes [32], [33]. Notably in CHC patients, there was a marked decrease of TGF-β serum levels after diet (Fig. 5A). We analyzed also another marker of inflammation, hyaluronic acid (HA) [34]. After NLCD levels of HA were significantly decreased in CHC patients, but not in NAFLD/NASH patients (Fig. 5A). Moreover, the IL-23 serum levels were evaluated because it has been demonstrated to be involved in the regulation of Th17 cells [35]. Before NLCD, IL-23 serum levels were higher than after treatment, even if no statistical significance was reached in both groups (Fig. 5B).

## Discussion

The importance of cholesterol in the HCV infection has been repeatedly described [2]–[5]. Thus, HCV infection is able to alter host lipid metabolism, leading to a hepatic steatosis. In this regard we administered a diet low in cholesterol to assess the impact that it could have on CHC patients. In fact, we describe that a Normocaloric Low Cholesterol diet is able to restore the immune homeostasis recovering the balance between Th17 and Treg cells in CHC patients, but not in those affected by NAFLD/NASH. Thus, our findings corroborate the importance of cholesterol metabolism during HCV infection.

Confirming several studies [36]–[38], we observed a remarkable higher frequency of Th17 cells in blood of CHC patients with respect to healthy individuals, and a slight increase in NAFLD/NASH subjects. Interestingly, NLCD was able to induce a consistent reduction of Th17-cell frequency in CHC patients, but not in our cohorts of NAFLD/NASH patients. As expected, after diet, we observed a decrease in serum levels of Th17-associated cytokines, i.e. IL-17 and IL-22, which was statistically significant only in CHC patients. Furthermore, we reported that Th17-cell frequency correlated, before and after NLCD, with ALT, an indicator of liver inflammation, but not with viral HCV replication or different HCV-genotypes (data not shown). Th17 cells represent a pro-inflammatory subset, while the Treg cells have an antagonistic effect; the deregulation of Th17/Treg balance has been associated with pathophysiological changes observed during the inflammation and the progression of autoimmune diseases [39], [40]. So we investigated if the modulation of Th17-cells observed after NLCD in CHC patients was also associated with a modulation of Treg cells and then with a change in Th17/Treg ratio. Before diet, we observed an imbalanced ratio between the Th17 and Treg cells confirm an inflammatory status of the patients studied [39]. In CHC patients after NLCD, we found a significant increase in Treg-cell frequency and in the Treg/Th17 ratio. Importantly, in NAFLD/NASH patients, no significant modulation of Th17/Treg balance, after NLCD, was observed (Fig. 3). These findings indicate a different modulation of the immune response between CHC and NAFLD patients; in fact, the Th17-cell frequency regulation seems to be related not only to the metabolic status, but mostly to the presence of the HCV.

Our study focused mainly on the frequency of Th17 cells, not only for their involvement in the progression of the disease, but especially because it has been described that their differentiation is regulated by cholesterol sensors and metabolic modulators, as LXRs [20], [21], [23], [24]. LXRs bind DNA as a heterodimer when joined to the RXR and regulates cholesterol and fatty acid metabolism genes, such as ABCA1 and SREBP1C [23]. On these evidences, we evaluated the LXRs activity by the study of the relative expression of LXRs and their target genes, SREBP-1c and ABCA-1. Specifically, it has been showed that LXR-induced Srebp-1 inhibits Il17 transcription binding the Il17 promoter, thus regulating T17-cell proliferation and differentiation [21]. After NLCD, we highlighted an increased mRNA-level expression of LXRs and of both their target genes in CHC patients, fact that, indirectly, supports an increased activity of the LXR nuclear receptors (Fig. 4). The increase of transcriptional levels of LXRβ in our cohorts of CHC patients was not expected, and, in our opinion, is quite intriguing. Further studies are needed to understand the role of LXRβ during HCV infection. The same panel of genes was evaluated in NAFLD/NASH patients after NLCD, obtaining a different modulation than in CHC subjects. This supports our idea that NLCD can counteract the HCV influence on LXRs activity, restoring the immune system homeostasis. Consequently, the Th17-cell frequency is heavily influenced by cholesterol metabolism, since the NLCD improves Th17/Treg balance, modulating LXRs and their target genes.

Finally, since TGF-β is known to be a regulator of Treg and Th17-cell differentiation [41]–[44], we looked at its serum concentration, in CHC and NAFLD/NASH patients before and after NLCD, taking into account that TGF-β, as well as HA, is a predictor of clinical worsening [32]–[34]. In particular, the key role of TGF-β in inflammation is proved by the development of several anti-TGF-β compounds for the treatment of a broad range of inflammatory, autoimmune diseases and cancer [45]. Therefore, in our experimental settings we evaluated TGF-β serum levels as an index of liver disease progression, also to circumvent the limitation the local ethics committee that allowed us to perform liver biopsy only on a limited group of patients with clinical signs of impaired liver function at the enrolment in the trial.

After NLCD, TGF-β serum concentration significantly decreased in CHC patients (almost 2 folds) and not in NAFLD/NASH patients. To note, the TGF-β is not produced exclusively by the Treg cells, in fact, within inflammatory microenvironment, it is secreted by Kupffer cells and activated hepatic stellate cells, indicating that its reduction not necessarily to reflect the frequency of Treg cells, but rather the improving the hepatic condition [46]. Moreover, CHC patients at the completion of the dietary regimen showed the same trend for the serum levels of HA. The down-regulation by the diet regimen of these two important validated biomarkers, for chronic liver inflammation, gains a statistical significance only in CHC patients (Fig. 5), leading us to hypothesize that NLCD could have a potential improvement in the onset and progression of disease. Furthermore, these data are also in keeping with the lowering of systemic inflammation indices, such as high-sensitivity C reactive protein (hs-CRP), after diet.

Regarding the biochemical parameters, after 30 days of NLCD in both groups, we have not observed a reduction in body weight, in the concomitant BMI levels, and in homeostasis model assessment (HOMA) index, but rather a significant decrease in LDL cholesterol, with a tendency towards reduction in total cholesterol and triglyceride levels. These results reflect our intention of modulating only the lipid metabolism with a NLCD in both CHC and NAFLD/NASH patients.

Further work in this area will no doubt yield insights into the impact of metabolic players on the shaping of the immune response. Many of the recent studies describe metabolic influence over T-cell fate and propose or included the initial evaluation of new molecular agonists and antagonists in animal models of autoimmune disease [12], [21], [39]. In conclusion, this study suggests that a NLCD is able to regulate the Th17/Treg balance, by LXRs activation, reducing the risk of an adverse outcome related to inflammation. A NLCD may result in a reduction in Th17 cells and recognized inflammatory complications of these cells in CHC including hepatic inflammation. Thus, our work supports the idea of a new approach in the management of chronic HCV-infected patients by changing lifestyle, promoting well-being and possibly hindering disease progression.

## Supporting Information

S1 CONSORT Checklist **CONSORT checklist.**
(PDF)

S1 Protocol **Trial protocol.**
(DOCX)
