# Supplementary material for: Normocaloric Low Cholesterol Diet Modulates Th17/Treg Balance in Patients with Chronic Hepatitis C Virus Infection
Source: PLoS One. 2014 Dec 22;9(12):e112346. doi: 10.1371/journal.pone.0112346 (PMC4273946; doi:10.1371/journal.pone.0112346)
Supplement: S1 Protocol — Trial protocol. (DOCX) [file pone.0112346.s002.docx]

**Synopsis**

| **Title** | Prospective clinical study on the role of the immune response, in relation to diet, in patients affected by either chronic hepatitis C virus (HCV) or Non Alcoholic Fatty Liver Disease (NAFLD) |
| --- | --- |
| **Background** | Chronic hepatitis C virus (HCV) infection infection and nonalcoholic fatty liver disease (NAFLD) are characterized by a spectrum of pathological conditions ranging from an early stage of inflammation and fibrosis up to more advanced disease conditions, such as hepatocellular carcinoma. The prevalence of NAFLD is between 10 and 25% of the population, with large differences in age and ethnic groups, while it is well known that HCV infection is a major cause of chronic liver disease in Western countries.  For both diseases the progression of liver damage is in close correlation with the lifestyle of patients (eg., nutrition, physical activity, ingestion of alcohol, etc.). In fact, it was shown that feeding imbalances may have implications in altering the normal immune functions of the subjects, suggesting that the metabolic and the immune systems are closely related to each other. Although it is well known the negative role of obesity on the progression of NAFLD and HCV liver diseases, the pathogenic mechanism underlying the alterations related to the immune response is not yet fully understood. Insulin resistance, altered lipid metabolism, lipid peroxidation, oxidative stress and mitochondrial alterations are pathogenic mechanisms that induce liver damage and its progression, both in NAFLD and in HCV infection.  Recent studies suggest that the evolution of viral infections and chronic inflammation in NAFLD are deeply influenced by CD4+ T helper cells expressing IL-17, defined as T helper 17 (Th17) cells. Broadening the knowledge on the role of diet in the course of NAFLD and HCV infection in the activation of Th17 cells and in the alteration of some of their functions, will allow to shed light on the pathogenic mechanisms underlying the progression of immune-mediated diseases. Moreover, this investigation will allow to understand whether Th17 cells may have a role in the diminished response to therapy in patients who have high cholesterol levels.  If the results will confirm our hypothesis, this study will provide useful informations for the clinical management of patients with both steatosis and chronic HCV infection. The data obtained can also be used for the development of new therapeutic strategies directed to modulate the antiviral immune response.  All patients will undergo clinical and instrumental assessment depending on the type of pathology. Patients will be required to follow a normocaloric low cholesterol diet for a period of 30 days.  The prospective clinical study does not present any form of additional risk for the patients and will be conducted in accordance with the principles established by the Declaration of Helsinki and with the standards of Good Clinical Practice (GCP). The study does not require any additional costs. |
| **Objectives**  **(Endpoints)** | ► **Primary objective**  To compare the immune response of patients with chronic HCV infection and NAFLD/NASH through the evaluation of lymphocyte subpopulations, particularly with regard to the lymphoid compartment Th17.  ► **Secondary objectives**   1. To evaluate the possible modulation of the Th17 frequency after administration of low-cholesterol diet for thirty days in the two studied populations. 2. To evaluate, in the same cohort of patients, of the biological mechanisms underlying the immune response of Th17 cells in relation to cholesterol metabolism, compared before and after low cholesterol diet. 3. To evaluate the lipid parameters (Total Cholesterol, Triglycerides, LDL, HDL) change after 30 days of diet |
| **Study Chair** | Prof. Clara Balsano, MD |
| **Contacts** | Prof. Vincenzo Barnaba, MD |
| **Locations** | Department of Internal Medicine, La Sapienza University, Rome Italy |
| **Target Disease** | Chronic HCV infection and NAFLD |
| **Eligibility** | ► Inclusion Criteria:   1. Age 40- 70 years old 2. Both gender 3. Diagnosis of chronic HCV infection or NAFLD   ► Exclusion criteria  Any pharmacological treatment at least 6 months before entering the study, liver cirrhosis, co-infection by hepatitis B virus, or human immunodeficiency virus infections, autoimmune diseases, and other relevant associated-diseases such as decompensated diabetes, kidney diseases, pulmonary diseases, tumors. |
| **Number of Subjects** | Sample size was determined on the basis of recent studies supporting the hypothesis that the Th17 cell frequency, the variable in our cohort of patients, has likely a mean of 3.2% ± 2.0. We hypothesized that diet can decrease the Th17 cell frequency to 2.0% ± 0.15, 90% power, significance level of 0.05; for this reason to perform the study, at least 24 patients per group are required. Thus, we enrolled 30 patients per group considering the possible dropout of about the 20% of them. |
| **Investigational Product** | ► Normocaloric low cholesterol diet |
| **Investigational product dose** | ► Treatment period : 30 days |
| **Methods** | Patients willing to participate will be screened after written informed consent is obtained. The patients were instructed to follow a Normocaloric Low Cholesterol Diet (1400 Kcal for females and 1800 Kcal for males) for a period of 30 days. Dietary compounds were distributed as follow: lipids 23% (cholesterol 185 mg/day), carbohydrates 50%, and proteins 27%. Dietary habits, before the study, were recorded for all enrolled patients.The patients were instructed to follow the same food recipe for meal preparation. The diet was provided by an expert nutritionist. Adherence to the diet was monitored by phone interview with a weekly frequency. Subjects were advised to maintain their normal living activity and sleeping patterns during the intervention period. Subjects were instructed to follow the diet on the day after baseline measurements (day 1). At the beginning (day 0, namely T0) and end (day 30, namely T30) of diet, blood samples were analyzed for general parameters including biochemical parameters, hematology, and coagulation values. |
| **Statistical**  **analysis** | Efficacy analysis was done on the full analysis set (the intention-to-treat population), comprising all patients treated. All patients who followed the diet were included in the safety analyses with descriptive statistics. For the comparison of demographic information and other baseline characteristics, continuous variables are analyzed using the independence-sample t test or ANOVA test. |
|  |  |
